# Zinc Uptake, Photosynthetic Efficiency and Oxidative Stress in the Seagrass *Cymodocea nodosa* Exposed to ZnO Nanoparticles

**DOI:** 10.3390/ma12132101

**Published:** 2019-06-29

**Authors:** Paraskevi Malea, Katerina Charitonidou, Ilektra Sperdouli, Zoi Mylona, Michael Moustakas

**Affiliations:** 1 Department of Botany, Aristotle University of Thessaloniki, GR-54124 Thessaloniki, Greece; 2 School of Agricultural Sciences, University of Thessaly, GR-38446 Volos, Greece; 3 Institute of Plant Breeding and Genetic Resources, Hellenic Agricultural Organisation-Demeter, Thermi, GR-57001 Thessaloniki, Greece

**Keywords:** adaptive response, hormetic response, hydrogen peroxide, marine angiosperms, non-photochemical quenching, photoprotective mechanism, plastoquinone pool, reactive oxygen species (ROS), redox state, zinc oxide nanoparticles

## Abstract

We characterized zinc oxide nanoparticles (ZnO NPs) by dynamic light scattering (DLS) measurements, and transmission electron microscopy (TEM), while we evaluated photosystem II (PSII) responses, Zn uptake kinetics, and hydrogen peroxide (H_2_O_2_) accumulation, in *C. nodosa* exposed to 5 mg L^−1^ and 10 mg L^−1^ ZnO NPs for 4 h, 12 h, 24 h, 48 h and 72 h. Four h after exposure to 10 mg L^−1^ ZnO NPs, we noticed a disturbance of PSII functioning that became more severe after 12 h. However, after a 24 h exposure to 10 mg L^−1^ ZnO NPs, we observed a hormetic response, with both time and dose as the basal stress levels needed for induction of the adaptive response. This was achieved through the reduced plastoquinone (PQ) pool, at a 12 h exposure, which mediated the generation of chloroplastic H_2_O_2_; acting as a fast acclimation signaling molecule. Nevertheless, longer treatment (48 h and 72 h) resulted in decreasing the photoprotective mechanism to dissipate excess energy as heat (NPQ) and increasing the quantum yield of non-regulated energy loss (Φ*_NO_*). This increased the formation of singlet oxygen (^1^O_2_), and decreased the fraction of open reaction centers, mostly after a 72-h exposure at 10 mg L^−1^ ZnO NPs due to increased Zn uptake compared to 5 mg L^−1^.

## 1. Introduction

The small size of nanoparticles (NPs) provides them with special physical and chemical properties that are not found in bulk materials allowing their utilization, among others, in agricultural products, catalysis, cosmetics, electronics, energy production, engineering, food industry, pharmaceutics and textiles [1,2,3,4,5]. 

Among the variety of metal NPs that are often used for marketable purposes, zinc oxide (ZnO) NPs are the most commonly used ones [6,7,8]. ZnO NPs, with their unique chemical and physical properties, such as high photostability, broad range of radiation absorption, high electrochemical coupling coefficient, and high chemical stability, are widely used in a diversity of applications, varying from paints to chemicals, from tires to ceramics, and from pharmaceuticals to agriculture [3,9]. ZnO NPs are specifically used in clothing, skin care products, anticancer medicines, sunscreens, coatings for solar cells, bottle coatings, and gas sensors [10]. 

The rapid expansion, due to their unique properties, and their release in the environment has raised considerable worries regarding manufactured NPs [11]. An essential aspect of the risk assessment of NPs is to understand their interactions with plants, a basic component of all ecosystems [11]. Thus, the extensive use of ZnO NPs has extended the requirements of research on their consequences on living organisms [6]. Environmental levels of ZnO-NPs were stated to be among 3.1–31 μg kg^−1^ soil and 76–760 μg L^−1^ water [12]. Manufactured NPs are unavoidably released into the soil and through streams, rivers, and sewage treatment they finally reach the sea [5]. Since NPs finally end in aquatic ecosystems, aquatic plants may be at higher risks than terrestrial. Thus, there is a need to evaluate the risks related to NP presence in aquatic ecosystems [12].

In photosynthesis, electron transport is mediated by photosystem I (PSI), and photosystem II (PSII) that work coordinately in the thylakoid membranes [13,14,15]. The most prone constituent of the photosynthetic apparatus to environmental stress is thought to be PSII [15,16,17,18]. Perturbations of PSII functionality resulted in declining the photosynthetic capacity, limiting the growth and development of plants, and reducing crop production [19,20]. Measuring PSII function by chlorophyll fluorescence imaging analysis is the most appropriate methodology to detect NPs-induced stress on plants [21,22,23] Metal oxides NPs alter photosynthetic efficiency by reducing energy transfer efficiency and quantum yield [5,21,23].

Reactive oxygen species (ROS) generated as byproducts in chloroplasts by the light reactions of photosynthesis are responsible for NPs induced toxicity [24], and thus the impact of NPs toxicity on plants can be also estimated by ROS production [22,25]. However, ROS generation can activate the plant’s defense mechanisms in order to cope with the oxidative stress damage [15,26,27,28,29]. 

*C. nodosa* (Ucria) Ascherson is a perennial, fast-growing seagrass that colonizes shallow waters and degraded environments [5,30]. It grows in coastal areas in vicinity to anthropogenic actions and has been proposed a suitable bio-indicator species [31]. In this species, cellular, physiological and biochemical measurable responses induced by various chemical stressors have been proposed to monitor environmental quality [32,33].

The objectives of our study were to investigate the relationship between ZnO NPs effects in *C. nodosa* with Zn uptake in order to understand their impact on seagrasses. We wanted to test whether exposure of seagrasses to NPs will be a dose dependent response (concentration- and time-dependent) or a hormetic response. We evaluated ZnO NPs effects on *C. nodosa* PSII photochemistry by chlorophyll fluorescence imaging analysis, and detected ROS generation as a byproduct of ZnO NP’s effects, linking also the ROS generation to PSII functionality.

## 2. Materials and Methods 

### 2.1. Plant Material

*C. nodosa* (Ucria) Ascherson plants were collected from the Gulf of Thessaloniki, Aegean Sea (40°33 N, 22°58 E), by their maximum leaf biomass production, at 0.7 m–1.0 m depth [34]. 

### 2.2. Experimental Conditions and Exposure to Zinc Oxide Nanoparticles

*C. nodosa* plants (leaves, orthotropic and plagiotropic rhizomes and roots) were kept in seawater aquaria that have a salinity of 36.9 psu, pH of 7.9, and dissolved oxygen of 5.9 mg L^−1^, using continuously aerated aquarium pumps as previously described [5].

Zinc oxide NPs with less than 50 nm particle sizes were purchased from Sigma-Aldrich (St. Louis, MO, USA). Stock solution of ZnO NPs in millique water (50 mg L^−1^), after sonication for 30 min, was stored in the dark at 4 °C [5]. ZnO NPs concentrations in natural waters stated to be among 76–760 μg L^−1^ [12] are below concentrations known to have environmental effects on aquatic organisms [35]. In preliminary experiments with 1 and 3 mg L^−1^ ZnO NPs, no effect was detected on PSII functionality. Thus, we applied 5 mg L^−1^ and 10 mg L^−1^ ZnO NPs, which is 7–13 times more the maximum levels of ZnO-NPs reported for water environments [12]. 

*C. nodosa* plants were exposed to 5 and 10 mg L^−1^ for 0 (control), 4 h, 12 h, 24 h, 48 h and 72 h. The two ZnO NPs concentrations were prepared with filtered (0.45 μm GF/C Whatman) seawater immediately before use. Control and treatment solutions were changed every 24 h. *C. nodosa* intermediate leaf blades (about 300 mm length) were used for chlorophyll fluorescence imaging, H_2_O_2_ imaging, and for Zn uptake measurements.

### 2.3. Zinc Oxide Nanoparticles Characterization

Primary particle size, and the morphological and structural characteristics of ZnO NPs were investigated by transmission electron microscopy (TEM). Samples were prepared by drop-casting dispersions of the NPs onto carbon-coated Cu grids after a 3 min sonication with an ultrasonic (VibraCell 400 W, Sonics & Materials Inc., Newtown, CT, USA), applying a microtip probe under intensity settings 4 [36]. Finally, TEM images were obtained with a Jeol JEM 1010 microscope (Jeol, Tokyo, Japan) [37].

Dynamic light scattering (DLS) analysis was used to define the size-distribution profile of ZnO NPs (5 mg L^−1^, 10 mg L^−1^ and 50 mg L^−1^). Zeta (ζ) potential measurements were conducted to assess the surface charge of the particles as described previously [5]. All measurements were performed in Milli-Q water after brief sonication at 25 °C [5]. Results are presented as means (±SD) of three measurements.

### 2.4. Zinc Determination

Intermediate blades after wet digestion were processed following the methodology described previously [38,39]. Zinc concentrations were determined by flame atomic absorption spectrophotometry (AAnalyst 400 FAAS, Perkin-Elmer, Waltham, MA, USA) with the procedure described in detail before [38,39].

### 2.5. Zinc Leaf Uptake Kinetics

Zinc leaf uptake kinetics was fitted to the Michaelis-Menten equation: (C*_max_* × *t*)/(K*_m_* + *t*), as described in detail previously [5]. Briefly, C represents Zn leaf concentration reached in time t, K*_m_* the time taken to reach half of the value of C*_max_*, and C*_max_* the maximum or saturation Zn concentration. The rate of the initial uptake (C*_max_*/2 × K*_m_*), the time needed to get equilibrium (T*_eq_*), the equilibrium concentration (C*_eq_*) and the mean rate of uptake (V*_c_*) were also estimated. Equilibrium concentration (C*_eq_*) is a concentration where the hourly increase is less than 1% compared to the previous hour [38,39,40,41]. The time required to reach equilibrium (T*_eq_*) was assessed as the time needed to get the C*_eq_*, and the mean rate of uptake (V*_c_*) was assessed as C*_eq_*/T*_eq_* [38,39]. Bioconcentration factor (BCF) was estimated as (C*_eq_* − C*_i_*)/C*_w_*, where C*_i_* is the initial Zn tissue concentration and C*_w_* is the Zn concentration in water [38,39]. 

### 2.6. Chlorophyll Fluorescence Imaging Analysis

An Imaging-PAM Chlorophyll Fluorometer (Walz, Effeltrich, Germany) was used for photosynthetic efficiency measurements as previously described [5]. In *C. nodosa* dark-adapted (15 min) leaf samples, we selected six areas of interest, and the allocation of absorbed light energy to photochemistry (Φ*_PSII_*), non-photochemical energy loss as heat (Φ*_NPQ_*), and non-regulated energy loss (Φ*_NO_*), were calculated as described previously [29]. Relative PSII electron transport rate (ETR), non-photochemical quenching (NPQ), and photochemical quenching (*q*_p_), were also measured [42]. 

Color-coded images, acquired with 200 μmol photons m^−2^ s^−1^, of Φ*_PSΙΙ_*, Φ*_NPQ_*, Φ*_NO_*, and the redox state of plastoquinone (PQ) pool (*q*_p_), are also presented.

### 2.7. Imaging of Hydrogen Peroxide Generation

For the estimation of H_2_O_2_ production, *C. nodosa* leaves were treated with 25 µM 2′,7′-dichlorofluorescein diacetate (Sigma) in the dark for 30 min, as described previously [43,44].

### 2.8. Statistical Analyses

Zinc leaf uptake kinetics data were analyzed using IBM Statistics SPSS^®^ 24 (New York, NY, USA). The significant differences on the fluorescence variables, between control and different incubation time in each concentration and between different concentrations at the same exposure time were tested at the 5% level of probability using t-test analysis (IBM Statistics SPSS^®^ 24). Modal analysis by using NORMSEP computer program was employed to estimate particle size (nm) distribution of ZnO NPs [45].

## 3. Results

### 3.1. Characterization of ZnO NPs

**Figure 1:**
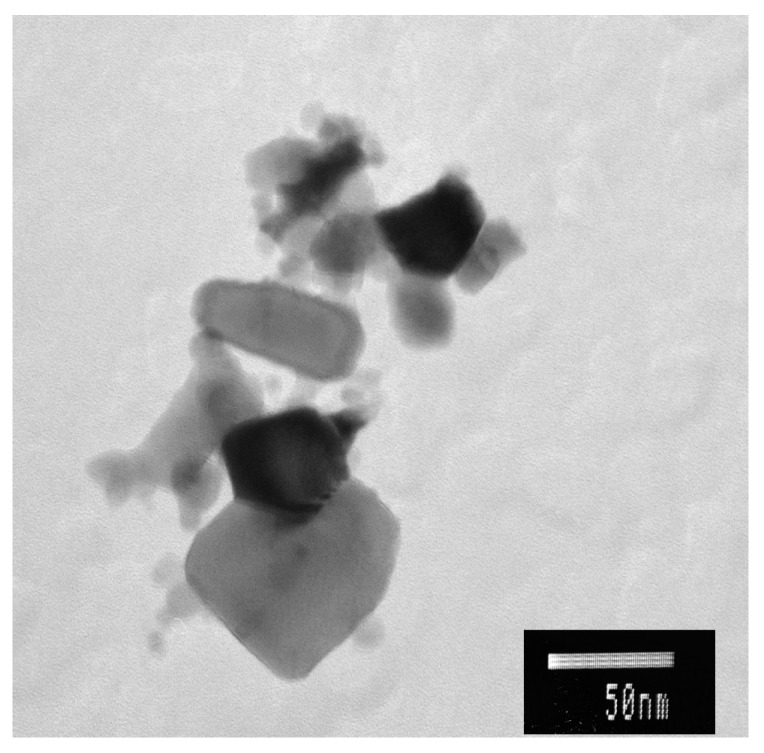
Transmission electron microscope (TEM) images of zinc oxide nanoparticles (ZnO NPs) stock solution (50 mg L^−1^).

**Figure 2:**
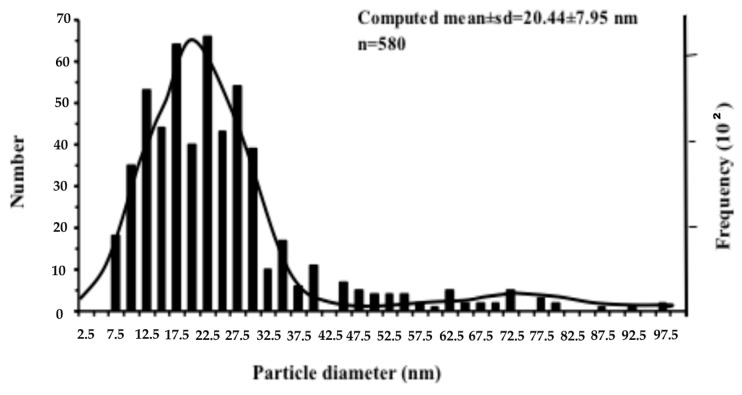
Distribution pattern of ZnO NPs from TEM micrographs.

The size and morphology of ZnO NPs was measured in stock solution by TEM (Figure 1). Modal analysis was used in data emerged from TEM micrographs, in order to determine the ZnO particle diameter distribution (Figure 2). A percentage of 92.8% of the particles was generally in agreement with manufacturer’s characteristics (size < 50 nm). The computed mean (±SD) NPs size group with the highest frequency was 20.44 nm ± 7.95 nm (Figure 2).

**Figure 3:**
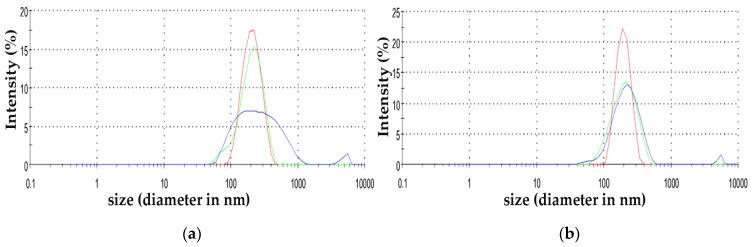
Size distribution by intensity of 5 mg L^−1^ ZnO NPs (**a**); and 10 mg L^−1^ ZnO NPs (**b**). Different colours indicate the replications.

The hydrodynamic size of 5 mg L^−^^1^, 10 mg L^−^^1^ and 50 mg L^−^^1^ (stock) ZnO NPs solutions, ranged from 220.6 nm to 225.0 nm (Table 1). The negative surface charge (ζ potential) ranged from −17.5 mV to −18.13 mV (Table 1). This similarity among the different NPs concentrations was indicative of the colloidal stability of the different populations. The size distribution by intensity of ZnO NPs is shown in Figure 3a,b.

### 3.2. Zinc Leaf Uptake Kinetics

**Figure 4:**
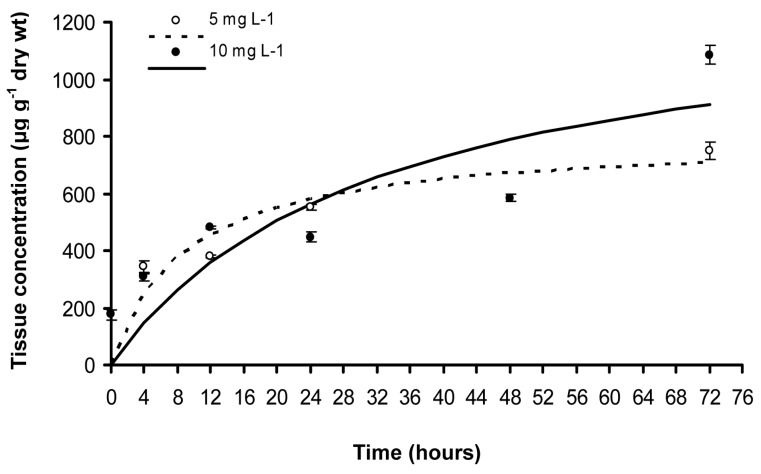
Kinetics of zinc uptake (μg g^−1^ dry weight) in *C. nodosa* leaf blades at 5 mg L^−1^ and 10 mg L^−1^ ZnO NPs ± SD (n = 3); dashed and bold lines are the uptake kinetics calculated using Michaelis-Menten equation.

Leaf Zn uptake at both ZnO NPs concentration displayed a time dependent variation (Figure 4). The uptake kinetics at both exposure concentrations was fitted to the Michaelis-Menten equation (r^2^:0.744 for 5 mg L^−1^, and 0.681 for 10 mg L^−1^, *p* < 0.01; Figure 4, Table 2). Zinc uptake of *C. nodosa* leaves increased more rapidly at the beginning of the experiment in the lower ZnO NPs solution (5 mg L^−1^), while it showed a higher and more than doubled initial rate [C*_max_*/(2 × K*_m_*)], and a higher mean rate (V*_c_*) in comparison to the 10 mg L^−1^ solution (Figure 4, Table 2). At 5 mg L^−1^ exposure concentration, the uptake reached the equilibrium concentration earlier (T*_eq_* = 28 h) than in the higher solution (42 h) (Table 2). However, both the maximum concentration (C*_max_*) and the equilibrium concentration (C*_eq_*) displayed their higher values at the higher ZnO NPs concentration (10 mg L^−1^) (Figure 4, Table 2). During the experiment, the highest uptake was observed after 72 h of exposure, at both ZnO NP treatments (748.7 ± 29.7 μg g^−1^ dry wt at 5 mg L^−1^ and 1086.0 ± 33.7 μg g^−1^ dry wt at 10 mg L^−1^) (Figure 4). Moreover, BCF value was higher at the lower exposure concentration (Table 2).

The fits correspond to a Michaelis-Menten equation: C = (C*_max_* × *t*)/(*K_m_* + *t*); C, in μg g^−1^ dry wt; *K_m_*, in hours; *t*, in hours; C*_eq_*, in μg g^−1^ dry wt; T*_eq_*, in hours; V*_c_*, concentration/hours. In parentheses, standard errors are given.

### 3.3. Allocation of Absorbed Light Energy in Leaf Blades of Cymodocea nodosa Exposed to ZnO NPs

**Figure 5:**
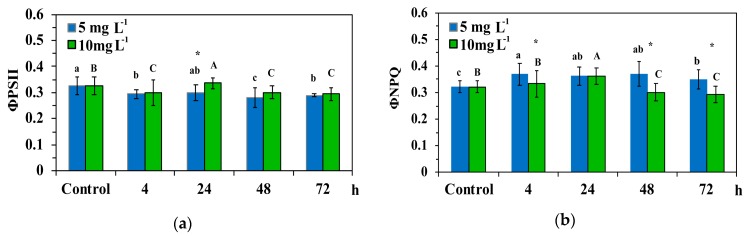
The quantum efficiency of PSII photochemistry (Φ*_PSΙΙ_*) (**a**); and the quantum yield of regulated non-photochemical energy loss as heat (Φ*_NPQ_*) (**b**), in control leaf blades of *C. nodosa* and in leaf blades exposed to 5 mg L^−1^ and 10 mg L^−1^ ZnO NPs for 4 h, 24 h, 48 h and 72 h. Columns with the same letter (lower case for 5 mg L^−1^ ZnO NPs and capitals for 10 mg L^−1^ ZnO NPs) are not statistically different (*p* < 0.05). An asterisk represents a significantly different mean of the same time treatment between 5 and 10 mg L^−1^ ZnO NPs (*p* < 0.05). Bars in columns represent standard deviation.

**Figure 6:**
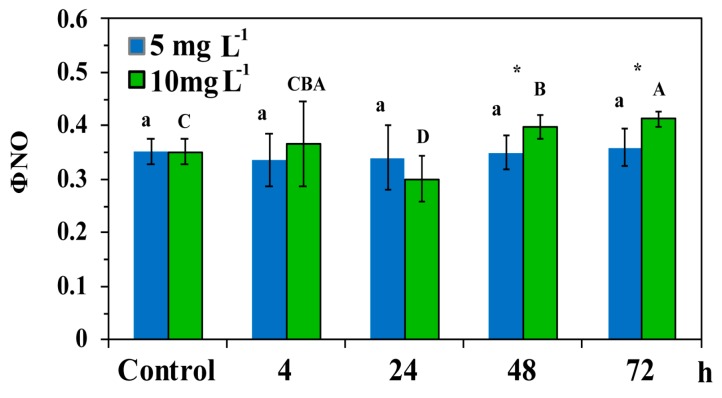
The quantum yield of non-regulated energy dissipated in PSII (non-regulated heat dissipation, a loss process due to PSII inactivity) (Φ*_NO_*) in control leaf blades of *C. nodosa* (*Cymodocea nodosa*) and in leaf blades exposed to 5 mg L^−1^ and 10 mg L^−1^ ZnO NPs for 4 h, 24 h, 48 h and 72 h. Symbol explanations as in Figure 5.

The allocation of absorbed light energy at PSII in leaf blades of *C. nodosa* exposed to ZnO NPs for 4 h, 24 h, 48 h and 72 h is shown in Figure 5 and Figure 6. Exposure to 5 and 10 mg L^−^^1^ ZnO NPs significantly decreased the quantum efficiency of PSII photochemistry (Φ*_PSΙΙ_*) compared to control values, with the exception of the 24 h treatment that Φ*_PSΙΙ_* increased at 10 mg L^−1^ ZnO NPs and did not differ compared to control values at 5 mg L^−^^1^ ZnO NPs (Figure 5a). Thus, at the 24-h treatment, Φ*_PSΙΙ_* in 10 mg L^−^^1^ ZnO NPs was higher than in 5 mg L^−^^1^ (Figure 5a).

The quantum yield of regulated non-photochemical energy loss (Φ*_NPQ_*) increased significantly at 5 mg L^−1^ ZnO NPs compared to control, while at 10 mg, L^−1^ increased after 24 h of exposure, but decreased at further exposure time (48 h and 72 h) (Figure 5b). Φ*_NPQ_* after 4 h, 48 h and 72 h exposure to the low concentration was significantly higher than in the high concentration (Figure 5b). 

The non-regulated energy loss (Φ*_NO_*) did not differ compared to control values after exposure to 5 mg L^−1^ ZnO NPs (Figure 6), but decreased compared to the control values after 24 h of exposure to the high NPs concentration, and increased at further exposure time (48 h and 72 h) (Figure 6). At 48 h and 72 h treatment Φ*_NO_* of *C. nodosa* leaf blades exposed to the high concentration was significantly higher than in the low concentration (Figure 6).

### 3.4. Electron Transport Rate and Non-Photochemical Quenching in Leaf Blades of Cymodocea nodosa Exposed to ZnO NPs

**Figure 7:**
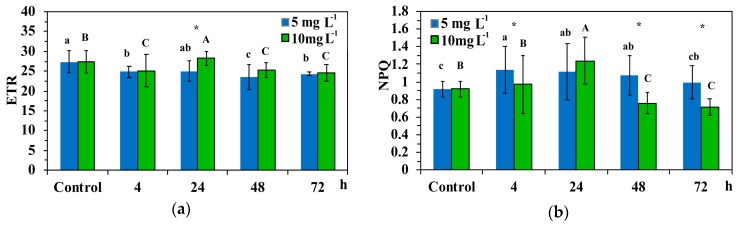
The relative electron transport rate of PSII (ETR) (**a**); and the non-photochemical quenching (NPQ) (**b**), in control leaf blades of *C. nodosa* and in leaf blades exposed to 5 mg L^−1^ and 10 mg L^−1^ ZnO NPs for 4 h, 24 h, 48 h and 72 h. Symbol explanations as in Figure 5.

The relative electron transport rate (ETR) of PSII decreased significantly at both ZnO NPs concentrations, with the exception of the 24 h exposure, where the ETR increased at the high concentration and did not differ compared to control values at the low concentration (Figure 7a). With the 24 h treatment, the ETR of *C. nodosa* leaf blades exposed to the high concentration was significantly higher than in the low concentration (Figure 7a). 

The non-photochemical quenching (NPQ) increased at the low concentration compared to control, while at the high concentration it increased after 24 h of exposure, but decreased significantly at further exposure time (48 h and 72 h) (Figure 7b). After 4 h, 48 h and 72 h treatment, the NPQ of *C. nodosa* leaf blades exposed to 5 mg L^−1^ was significantly higher than 10 mg L^−1^ (Figure 7b). 

### 3.5. The Redox State of Plastoquinone Pool of Cymodocea nodosa Leaf Blades Exposed to ZnO NPs

**Figure 8:**
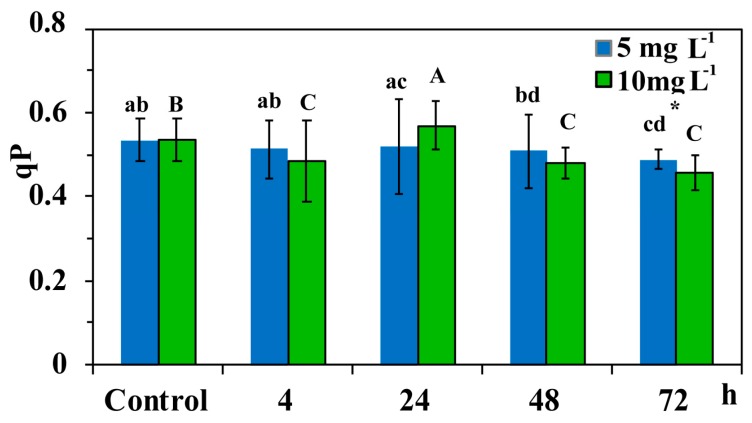
The photochemical quenching (*q*_p_) in control leaf blades of *C. nodosa* and in leaf blades exposed to 5 mg L^−1^ and 10 mg L^−1^ ZnO NPs for 4 h, 24 h, 48 h and 72 h. Symbol explanations as in Figure 5.

The redox state of plastoquinone (PQ) pool (a measure of PSII open reaction centers) (*q*_p_), did not differ compared to control values after 4 h, 24 h, and 48 h exposure to 5 mg L^−1^ ZnO NPs but reduced after 72 h (Figure 8). PQ pool reduced compared to control values after 4 h, 48 h and 72 h exposure to 10 mg L^−1^ ZnO, but increased after a 24 h exposure (Figure 8). After a 72 h exposure of *C. nodosa* to ZnO NPs, the PQ pool was more oxidized at 5 mg L^−1^ than at 10 mg L^−1^, but less than controls (Figure 8).

### 3.6. Chlorophyll Fluorescence Images of Cymodocea nodosa Leaf Blades Exposed to ZnO NPs

**Figure 9:**
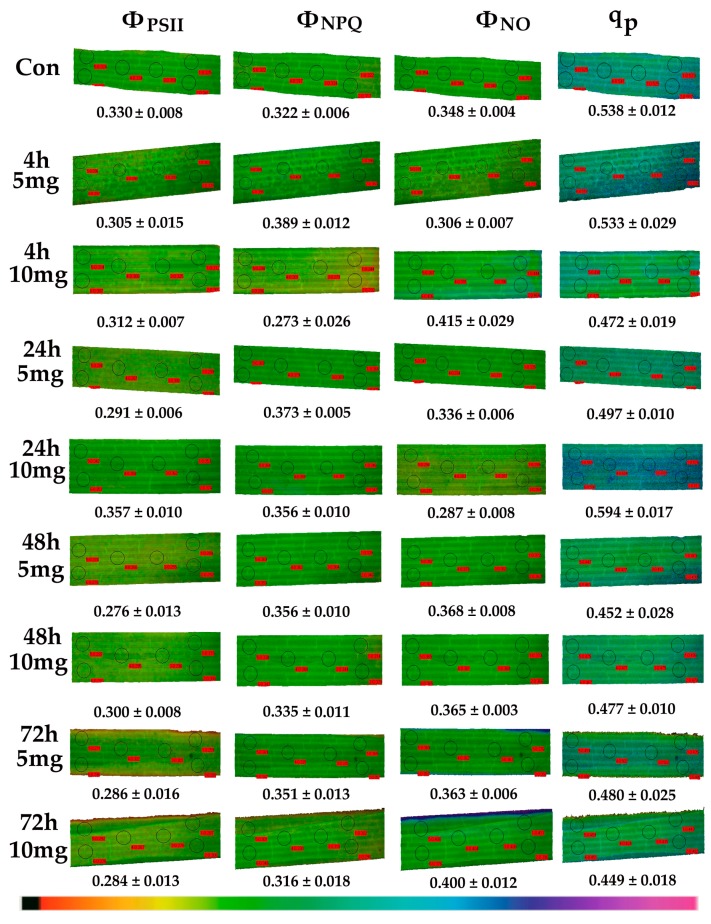
Representative chlorophyll fluorescence images at 200 μmol photons m^−2^ s^−1^ actinic light of Φ*_PSΙΙ_*, Φ*_NPQ_*, Φ*_NO_*, and *q*_p_; of *C. nodosa* control leaf blades and leaf blades exposed to 5 mg L^−^^1^ and 10 mg L^−^^1^ ZnO NPs for 4 h, 24 h, 48 h and 72 h. The colour code depicted at the bottom of the images ranges from black (pixel values 0.0) to purple (1.0). The six areas of interest are shown. Average values are presented for each photosynthetic parameter.

**Figure 10:**
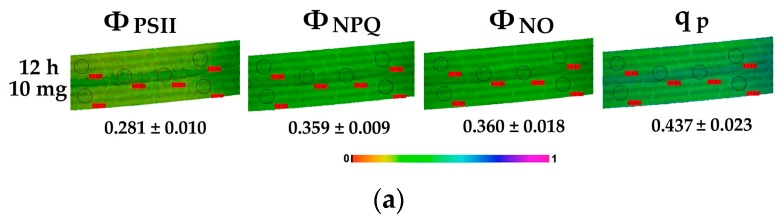

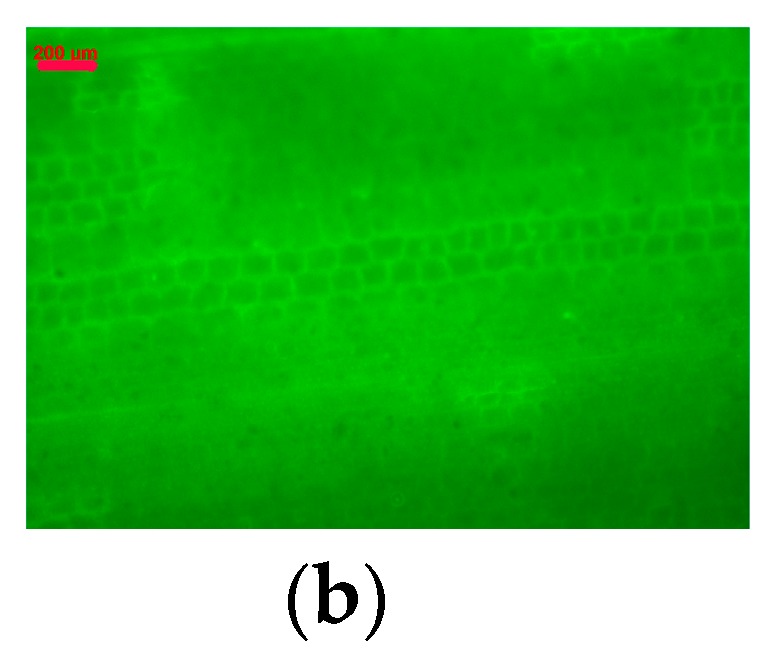
Representative chlorophyll fluorescence images after 5 min illumination at 200 μmol photons m^−2^ s^−1^ actinic light of Φ*_PSΙΙ_*, Φ*_NPQ_*, Φ*_NO_*, and *q*_p_ of *C. nodosa* leaf blades exposed to 10 mg L^−^^1^ ZnO NPs for 12 h. The colour code depicted at the bottom of the images ranges from 0.0 to 1.0. The average values are presented for each photosynthetic parameter (**a**). Below is the representative pattern of H_2_O_2_ production in a *C. nodosa* leaf blade exposed for 12 h to 10 mg L^−1^ ZnO NPs. Scale bare: 200 µm. Increased H_2_O_2_ content is indicated by light green colour (**b**).

We did not detect any spatial heterogeneity of Φ*_PSII_*, Φ*_NPQ_*, Φ*_NO_*, and *q*_P_ images in the control leaf blades of *C.* *nodosa* measured at 200 μmol photons m^−2^ s^−1^ actinic light (Figure 9). In addition, exposure to both NPs concentrations for 4 h, 24 h, 48 h and 72 h did not significantly alter these patterns (Figure 9). A temporal heterogeneity was observed at the images of *q*_P_ and Φ*_NPQ_* (Figure 9). The lowest Φ*_PSΙΙ_* with simultaneous low *q*_P_ values was observed after a 12 h exposure at the high concentration (Figure 10a). At the same time, the highest levels of H_2_O_2_ were detected (Figure 10b). Immediately after this, we noticed that the 24 h exposure to 10 mg L^−1^ ZnO NPs resulted in the highest Φ*_PSΙΙ_* values with the highest *q*_P_ values, both of them being higher than the control values (Figure 9). A parallel decreased Φ*_NO_* was detected (Figure 9).

### 3.7. Imaging of Hydrogen Peroxide Production After Exposure of Cymodocea nodosa Leaf Blades to ZnO NPs 

**Figure 11:**
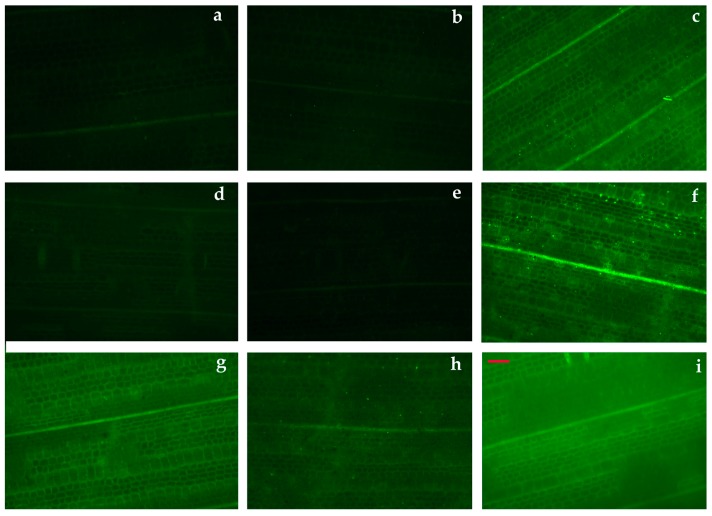
Representative patterns of H_2_O_2_ production in *C. nodosa* control leaf blades and after exposure to 5 mg L^−^^1^ and 10 mg L^−^^1^ ZnO NPs, as indicated by the fluorescence of H_2_DCF-DA. The H_2_O_2_ real-time generation in control leaf blade (**a**); after a 4-h exposure to 5 mg L^−^^1^ (**b**); after a 4-h exposure to 10 mg L^−^^1^ (**c**); after a 24-h exposure to 5 mg L^−^^1^ (**d**); after a 24-h exposure to 10 mg L^−^^1^ (**e**); after a 48-h exposure to 5 mg L^−^^1^ (**f**); after a 48-h exposure to 10 mg L^−^^1^ (**g**); after a 72-h exposure to 5 mg L^−^^1^ (**h**); and after a 72-h exposure to 10 mg L^−^^1^ (**i**). Scale bare: 200 µm. A higher H_2_O_2_ content is indicated by light green colour.

No noteworthy quantities of H_2_O_2_ could be noticed in the control leaf blades of *C. nodosa* (Figure 11a). Exposure at the low concentration for 4 h did not result in any change to H_2_O_2_ production (Figure 11b), while the same exposure time at the high concentration, resulted in increased production of H_2_O_2_ (Figure 11c). After a 24-h exposure to 5 mg L^−1^ ZnO NPs, H_2_O_2_ levels were the same as the control ones (Figure 11d), while they also dropped at 10 mg L^−1^ and could not be detected at all (Figure 11e). Later, after a 48-h exposure to ZnO NPs, there was an increase in the accumulation of H_2_O_2_ being higher at 5 mg L^−1^ (Figure 11f), than at 10 mg L^−1^ ZnO NPs (Figure 11g). However, after a 72-h exposure to ZnO NPs the accumulation of H_2_O_2_ decreased at 5 mg L^−1^ (Figure 11h), but increased at 10 mg L^−1^ ZnO NPs (Figure 11i). The highest H_2_O_2_ accumulation was detected after a 12-h exposure to 10 mg L^−1^ ZnO NPs (Figure 10b) and the lowest after a 24-h exposure to 10 mg L^−1^ (Figure 11e).

## 4. Discussion

Previously, extensive literature survey has demonstrated both the positive and detrimental impacts of NPs on terrestrial and aquatic plants, which are due to size and type of NPs (especially their specific surface area) and the plant species [5,7,8,12,22,25]. Toxicity of ZnO NPs is determined to be due to the dissolution, release and uptake of free Zn ions, but specific nanoparticulate effects may be hard to unravel from effects due to free zinc ions [46,47]. Thus, ZnO NPs effects in *C. nodosa* were correlated to both applied ZnO NPs concentration and to Zn uptake. 

In *C. nodosa* cellular, physiological and biochemical measurable responses (biomarkers) to metallic elements (e.g., Cd, Cr, Cu, Ni) have been proposed as early warning signals of alterations in seawater quality [32,33,38]. However, there has been little consideration of the seagrasses, especially *C. nodosa,* as the test material in evaluating the effects of metal oxide nanoparticles [5], despite the fact that coastal ecosystems are expected to be the destination of the majority of the nanoparticles, mainly ZnO and TiO_2_ NPs, discharged by industry [46]. NPs are released into aquatic environment either by direct uses or wastewater plant effluents [47,48,49].

Photosynthetic organisms via the process of photosynthesis, transform the light energy into chemical energy with the collaboration of PSII and PSI, while the most susceptible constituent of the photosynthetic apparatus to environmental stresses is believed to be PSII [15,16,17,18,50]. PSII functionality estimated by chlorophyll fluorescence imaging has been considered as the most suitable method to identify NPs toxicity effects on plants [5,21,22]. Exposure of plants to NPs can have positive or negative effects on the light reactions of photosynthesis [51]. 

At the beginning of the experiment, Zn uptake kinetics at 5 mg L^−1^ ZnO NPs with a more than twice initial rate and higher mean rate (V*_c_*) than 10 mg L^−1^ (Figure 4, Table 2), resulted in a significant lower quantum efficiency of PSII photochemistry (Φ*_PSΙΙ_*) compared to 10 mg L^−1^, after a 24 h exposure (Figure 5a). However, at 5 mg L^−1^ exposure concentration, Zn uptake reached the equilibrium concentration earlier (T*_eq_* = 28 h) than at 10 mg L^−1^ (T*_eq_* = 42 h), resulting in a significantly higher regulated non-photochemical energy loss as heat (Φ*_NPQ_*) after 48 h and 72 h exposure, compared to 10 mg L^−1^ (Figure 5b). This lower Φ*_NPQ_* at 10 mg L^−1^ ZnO NPs, resulted in significantly higher non-regulated energy loss (Φ*_NO_*) of *C. nodosa* leaf blades exposed to 10 mg L^−1^ for 48 h and 72 h (Figure 6), since there was no difference in Φ*_PSΙΙ_* (Figure 5a). The significantly increased Φ*_NO_* (Figure 6) implies higher singlet oxygen (^1^O_2_) production. Φ*_NO_* consists of chlorophyll fluorescence internal conversions and intersystem crossing, indicative of ^1^O_2_ formation via the triplet state of chlorophyll (^3^chl*) [42,52,53,54]. The increased ^1^O_2_ formation, mostly after 72 h exposure to 10mg L^−1^ ZnO NPs, was due to an increased Zn uptake compared to 5 mg L^−1^ (Figure 4).

A reduced photosynthetic efficiency, measured as the maximum quantum efficiency of PSII (*F*_v_/*F*_m_), and the redox state of plastoquinone (PQ) pool (*q*_p_), was also observed previously due to increased Zn accumulation [7]. Furthermore, ZnO NPs treatments enhanced generation of H_2_O_2_ [7]. A relationship between closed reaction centers (*q*_p_) and increased H_2_O_2_ generation was noticed (Figure 9, Figure 10, Figure 11). The PQ pool is considered as the component integrated in plant antioxidant defense [55], which at a 12 h of exposure mediated the generation of chloroplastic H_2_O_2_ [22], acting as a fast acclimation-signaling molecule [55,56]. 

After 12 h exposure of *C. nodosa* to 10 mg L^−^^1^ ZnO NPs, leaf Zn uptake was higher than in 5 mg L^−1^ (Figure 4), resulting in the lower Φ*_PSΙΙ_* and the lowest *q*_p_ values (Figure 10a). At the same time, an increased accumulation of H_2_O_2_ was detected in the leaves of *C. nodosa* (Figure 10b). Closed reaction centers (*q*_p_) indicate excess photon supply and associated ROS production [57,58,59]. This photosynthesis derived H_2_O_2_ is moving throughout leaf veins, serving as a signaling molecule and triggering a stress-defense response [22,35,44,55,56,60,61]. Thus, this stress-defense response triggered a significant increase of the fraction of open PSII reaction centers (*q*_p_) (Figure 8) and a significant increase of Φ*_PSΙΙ_* (Figure 5a) and ETR (Figure 7a) observed after 24 h exposure of *C. nodosa* to 10 mg L^−1^ ZnO NPs; while at the same time, H_2_O_2_ generation decreased to control levels (Figure 11e). However, we have to emphasize that antibacterial activity of ZnO and ZnO NPs is due to ROS generation [62,63], which also orchestrates a regulatory action at various plant developmental stages [64].

Hormesis has been extensively documented in plants, revealing that biphasic dose-responses occur commonly [65,66]. Thus, at low-level stress, plants are activating responses at the cellular and molecular level that enhance adaptation and plant tolerance [65,66]. The photoprotective mechanism of non-photochemical quenching (NPQ) which is closely related to ROS, follows a biphasic dose-response pattern typical of hormesis [67]. NPQ in *C. nodosa* leaf blades exposed to 10 mg L^−1^ ZnO NPs depicts a hormetic response. A hormetic response suggests that a basal stress level is needed for adaptive responses [68,69]. This basal stress level was 10 mg L^−1^ ZnO NPs and the time required for the induction of this mechanism was 24 h exposure. 

## Figures and Tables

**Table 1:** Hydrodynamic size (±SD) (diameter in nm) and zeta potential (±SD) (mV) values of the ZnO NPs (n = 3).

Formulation	Size	Zeta Potential
ZnO 50 mg L^−1^	221.0 ± 7.2	−17.93 ± 0.11
ZnO 10 mg L^−1^	225.0 ± 11.2	−17.50 ± 2.15
ZnO 5 mg L^−1^	220.6 ± 5.0	−18.13 ± 0.55

**Table 2:** Kinetics of Zn accumulation in *C. nodosa* leaf blades exposed to 5 mg L^−1^ and 10 mg L^−1^ ZnO NPs.

Parameter	5 mg L^−1^	10 mg L^−1^
C*_max_*	795.4 (±178.9)	1316.7 (±614.7)
*K_m_*	8.8 (±6.7)	32.1 (±34.2)
C*_max_*/(2 × *K_m_*)	44.950	20.507
r^2^	0.744 *	0.681 *
C*_eq_*	573.272	761.263
T*_eq_*	28	42
V*_c_*	20.473	18.125
BCF	79.588	58.593

* *p* < 0.01.
